# Respiratory sound classification for crackles, wheezes, and rhonchi in the clinical field using deep learning

**DOI:** 10.1038/s41598-021-96724-7

**Published:** 2021-08-25

**Authors:** Yoonjoo Kim, YunKyong Hyon, Sung Soo Jung, Sunju Lee, Geon Yoo, Chaeuk Chung, Taeyoung Ha

**Affiliations:** 1 Division of Pulmonology and Critical Care Medicine, Department of Internal Medicine, College of Medicine, Chungnam National University, Daejeon, 34134 Republic of Korea; 2 Division of Medical Mathematics, National Institute for Mathematical Sciences, Daejeon, 34047 Republic of Korea; 3 Clinical Research Division, National Institute of Food and Drug Safety Evaluation, Cheongju-si, Chungcheongbuk-do Republic of Korea; 4 Infection Control Convergence Research Center, Chungnam National University School of Medicine, Daejeon, 35015 Republic of Korea

**Keywords:** Diagnosis, Health services

## Abstract

Auscultation has been essential part of the physical examination; this is non-invasive, real-time, and very informative. Detection of abnormal respiratory sounds with a stethoscope is important in diagnosing respiratory diseases and providing first aid. However, accurate interpretation of respiratory sounds requires clinician’s considerable expertise, so trainees such as interns and residents sometimes misidentify respiratory sounds. To overcome such limitations, we tried to develop an automated classification of breath sounds. We utilized deep learning convolutional neural network (CNN) to categorize 1918 respiratory sounds (normal, crackles, wheezes, rhonchi) recorded in the clinical setting. We developed the predictive model for respiratory sound classification combining pretrained image feature extractor of series, respiratory sound, and CNN classifier. It detected abnormal sounds with an accuracy of 86.5% and the area under the ROC curve (AUC) of 0.93. It further classified abnormal lung sounds into crackles, wheezes, or rhonchi with an overall accuracy of 85.7% and a mean AUC of 0.92. On the other hand, as a result of respiratory sound classification by different groups showed varying degree in terms of accuracy; the overall accuracies were 60.3% for medical students, 53.4% for interns, 68.8% for residents, and 80.1% for fellows. Our deep learning-based classification would be able to complement the inaccuracies of clinicians' auscultation, and it may aid in the rapid diagnosis and appropriate treatment of respiratory diseases.

## Introduction

The stethoscope has been considered as an invaluable diagnostic tool ever since it was invented in the early 1800s. Auscultation is non-invasive, real-time, inexpensive, and very informative^1–3^. Recent electronic stethoscopes have rendered lung sounds recordable, and it facilitated the studies of automatically analyzing lung sounds^4,5^. Abnormal lung sounds include crackles, wheezes, rhonchi, stridor, and pleural friction rubs (Table 1). Crackles, wheezes and rhonchi are the most commonly found among them, and detecting those sounds greatly aids the diagnosis of pulmonary diseases^6,7^. Crackles, which are short, explosive, and non-musical, are produced by patients with parenchymal lung diseases such as pneumonia, interstitial pulmonary fibrosis (IPF), and pulmonary edema^1,8,9^. Wheezes are musical high-pitched sounds associated with airway diseases such as asthma and chronic obstructive pulmonary disease (COPD). Rhonchi are musical low-pitched sounds similar to snores, usually indicating secretions in the airway, and are often cleared by coughing^1^.

**Table 1:** Classification of abnormal lung sounds.

	Mechanism of sound production	Location	Characteristics	Acoustics	Related diseases
Fine crackles	Unrelated to secretions	Peripheral lung	Discontinuous High-pitched Inspiratory	Rapidly dampened wave deflection Frequency: about 650 Hz Shorter duration (about 5 ms)	Interstitial lung fibrosis Pneumonia Congestive heart failure
Coarse crackles	Intermittent airway opening, related to secretions	Peripheral lung	Discontinuous Low-pitched Inspiratory	Rapidly dampened wave deflection Frequency about 350 Hz Longer duration (about 15 ms)	Same as fine crackles but usually more advanced disease
Wheezes	Narrowed airway Flow limitation	Bronchi	Continuous High-pitched Expiratory > Inspiratory	Sinusoid Frequency > 100–5000 Hz Duration > 80 ms	Asthma COPD Tumor Foreign body
Rhonchus	Rupture of fluid films of secretions Airway wall vibrations	Bronchi	Continuous Low-pitched Expiratory > Inspiratory	Sinusoid Frequency about 150 Hz Duration > 80 ms	Bronchitis Pneumonia
Stridor	Narrowed airway	Larynx, Trachea	Continuous High-pitched Inspiratory	Sinusoid Frequency > 500 Hz	Epiglottitis After extubation Foreign body
Pleural friction rub	Pleural inflammation	Chest wall	Continuous Low-pitched Inspiratory and expiratory	Rhythmic succession of short sounds Frequency < 350 Hz Duration > 15 ms	Pleurisy Pericarditis Pleural tumor

Although auscultation has many advantages, the ability to analyze respiratory sounds among clinicians varies greatly depending on individual clinical experiences^6,10^. Salvatore et al. found that hospital trainees misidentified about half of all pulmonary sounds, as did medical students^11^. Melbye et al. reported significant inter-observer differences in terms of discriminating expiratory rhonchi and low-pitched wheezes from other sounds, potentially compromising diagnosis and treatment^12^. These limitations of auscultation raised the need to develop a standardized system that can classify accurately respiratory sounds using artificial intelligence (AI). AI-assisted auscultation can help a proper diagnosis of respiratory disease and identify patients in need of emergency treatment. It can be used to screen and monitor patients with various pulmonary diseases including asthma, COPD and pneumonia^13,14^.

Recently, deep learning is widely applied to some medical fields including a chest x-ray or electroencephalogram analysis^15,16^. There are several published studies on AI-assisted auscultation of heart and lung sounds^13,17–20^. AI was used to distinguish different murmurs and diagnose valvular and congenital heart diseases^21^. Auscultation of the lung is different from that of the heart in some aspects. First, the lungs are much larger than the heart; and lung sounds should be recorded at multiple sites of both lungs for an accurate analysis. Second, the quality of lung sound is easily affected by the patient’s effort to breathe.

There are several studies that tried to automatically analyze and classify respiratory sounds^15,22–30^. An interesting study quantified and characterized lung sounds in patients with pneumonia for generating acoustic pneumonia scores^22^. The sound analyzer was helpful for detecting pneumonia with 78% sensitivity and 88% specificity. In another study, crackles and wheezes in 15 children were applied to feature extraction via time–frequency/scale analysis; the positive percent agreement was 0.82 for crackle and 0.80 for wheezing^23^. Tomasz et al. used neural network (NN)-based analysis to differentiate four abnormal sounds (wheezes, rhonchi, and coarse and fine crackles) in 50 children^18^. Intriguingly, the results showed that the NN F1-score was much better than that of doctors. Gorkem et al. used a support vector machine (SVM), the k-nearest neighbor approach, and a multilayer perceptron to detect pulmonary crackles^24^. Gokhan et al. proposed the automatic detection of the respiratory cycle and collected synchronous auscultation sounds from COPD patients^28,31,32^. Interestingly, they demonstrated that deep learning is useful for diagnosing COPD and classifying the severity of COPD with significantly high-performance rates^28,29^.

Another study employed a sound database of the international conference on biomedical and health informatics (ICBHI) 2017 for classifying lung sounds using a deep convolutional NN. They converted the lung sound signals to spectrogram images by using the time–frequency method, but the accuracy was relatively low (about 65%)^25^. There are many feature extractors with CNN classifiers including inception V3, DenseNet201, ResNet50, ResNet101, VGG16, and VGG19^33–38^. In this study, we tried to combine pre-trained image feature extraction from time-series, respiratory sound, and CNN classification. We also compared the performances of these feature extractors.

Although this field has been being actively studied, it is still in its infancy with significant limitations. Many studies enrolled patients of a limited age group (children only), and some studies analyzed the sounds of a small numbers of patients. The studies that used the respiratory sounds of the ICBHI 2017 or the R.A.L.E. Repository database have a limitation in types of abnormal sounds. The ICBHI database contained crackles and wheezes only, and R.A.L.E. database lacked rhonchi^39^.

In this study, we aimed to classify normal respiratory sounds, crackles, wheezes, and rhonchi. We made a database of 1,918 respiratory sounds from adult patients with pulmonary diseases and healthy controls. Then we used transfer learning and convolutional neural network (CNN) to classify those respiratory sounds. We tried to combine pre-trained image feature extraction from time-series, respiratory sound, and CNN classification. In addition, we measured how accurately medical students, interns, residents, and fellows categorized breathing sounds to check the accuracy of auscultation classification in real clinical practice.

## Results

### The general characteristics of the enrolled patients and the collected lung sounds

We recorded 2840 sounds and the respiratory sounds were evaluated by three pulmonologists independently and classified. Then we made a respiratory sound database contained 1222 normal sounds (63.7%) and 696 abnormal sounds (36.3%) including 297 crackles (15.5%), 298 wheezes (15.5%), and 101 rhonchi (5.3%). Our database of classified sounds was consisted of 1918 sounds from 871 patients in the clinical field. Their demographic and clinical characteristics are presented in Table 2. The mean patient age was 67.7 (± 10.9) years and 64.5% of patients were male. Sounds were collected from patients with pneumonia, IPF, COPD, asthma, lung cancer, tuberculosis, and bronchiectasis, as well as healthy controls. The proportions of COPD and asthma patients were 21% and 12.3% respectively, the pneumonia proportion 11.1%, the IPF proportion 8.0%, and the healthy control proportion 5.9%. The location of auscultation was most common in both lower lobe fields. (Table 2).

**Table 2:** Characteristics of respiratory sound database.

871 cases (recording sounds: n = 1918)	Characteristics
Age, mean (years)	67.7 ± 10.9
Sex	Male: 562 (64.5%), Female: 309 (35.5%)
Diagnosis	Pneumonia: 11.1%, IPF: 4.9%, COPD: 21%, Asthma: 12.3%, Lung cancer/mass: 13.1%, Healthy: 5.9%, Tuberculosis: 5.4%, Bronchiectasis: 4.4% ILD except IPF: 8.0%, ACO: 0.3% ETC: 13.5%
Respiratory sounds	Normal: 1222 (63.7%), Crackles: 297(15.5%), Wheezes: 298 (15.5%), Rhonchi 101 (5.3%)
Auscultation location	RULF: 10.4%, RMLF: 6.9%, RLLF: 34.8% LULF: 16.3%, LMLF: 9.0%, LLLF: 21.7%, Neck 0.9%

*RULF* right upper lobe field, *RMLF* right middle lobe field, *RLLF* right lower lobe field, *LULF* left upper lobe field, *LMLF* left middle lobe field, *LLLF* left lower lobe field, *COPD* chronic obstructive pulmonary disease, *ILD* interstitial lung disease, *IPF* idiopathic pulmonary disease, *ACO* asthma-COPD overlap.

### Performance of AI-assisted lung sound classification

#### Discriminating normal sounds from abnormal sounds (crackles, wheezes, and rhonchi)

**Figure 1:**
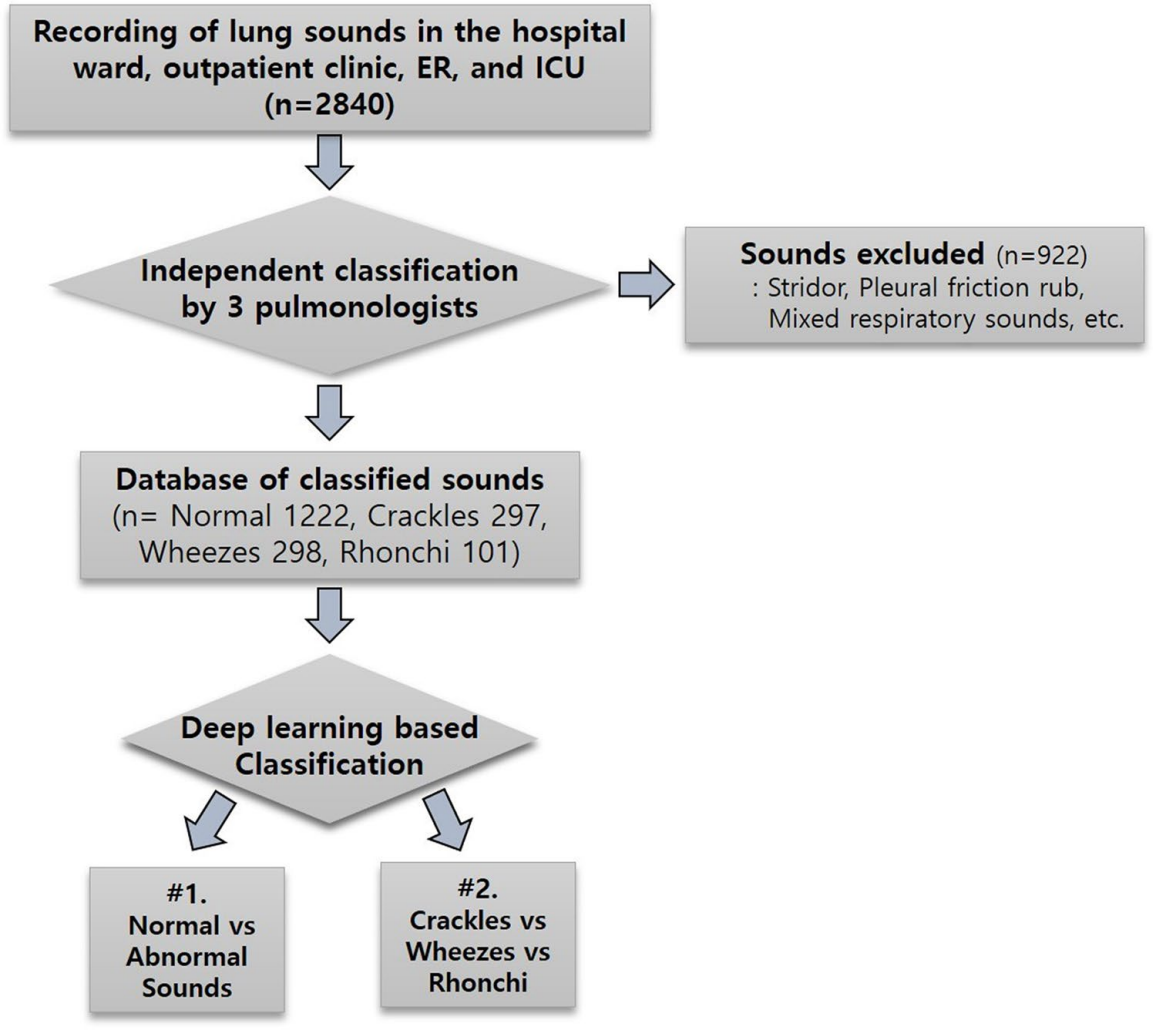
Scheme of the classification of respiratory sounds using deep learning. Lung sounds database contains normal sounds, crackles, wheezes, and rhonchi. Deep learning was used for two types of classification: The first step is the discriminating normal sounds from abnormal sounds. The second is to categorize abnormal sounds into crackles, wheezes, and rhonchi. (ER: Emergency room, ICU: intensive care unit).

**Figure 2:**
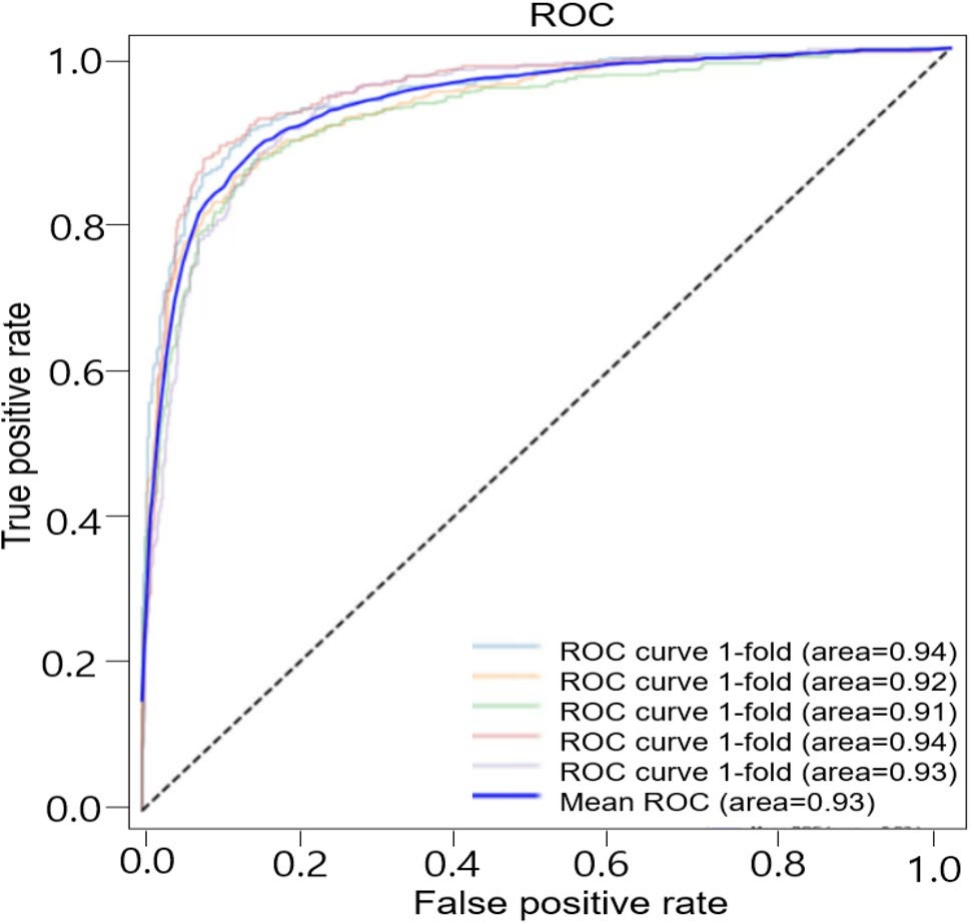
ROC of the model for discrimination of abnormal lung sounds. Each plot illustrates the ROC of the algorithm on the independent testing set for abnormal lung sounds, with AUC of 0.93.

In clinical settings, distinguishing abnormal breathing sounds and normal sounds is very important in screening emergency situations and deciding whether to perform additional tests. Our sound database included 1222 normal sounds and 696 abnormal sounds. We first checked how accurately our deep-learning based algorithm can classify abnormal respiratory sounds from normal sounds (Fig. 1). The precision, recall, and F1 scores for abnormal lung sounds were 84%, 80%, and 81% respectively (Table 3). The accuracy was 86.5% and the mean AUC was 0.93 (Fig. 2).

**Table 3:** The averages of Precision, Recall and F1 score in discriminating normal sounds from abnormal sounds.

	Precision	Recall score	F1 score
Normal	0.88	0.91	0.89
Abnormal	0.84	0.80	0.81

#### Categorization of abnormal sounds into crackles, wheezes, and rhonchi

**Figure 3:**
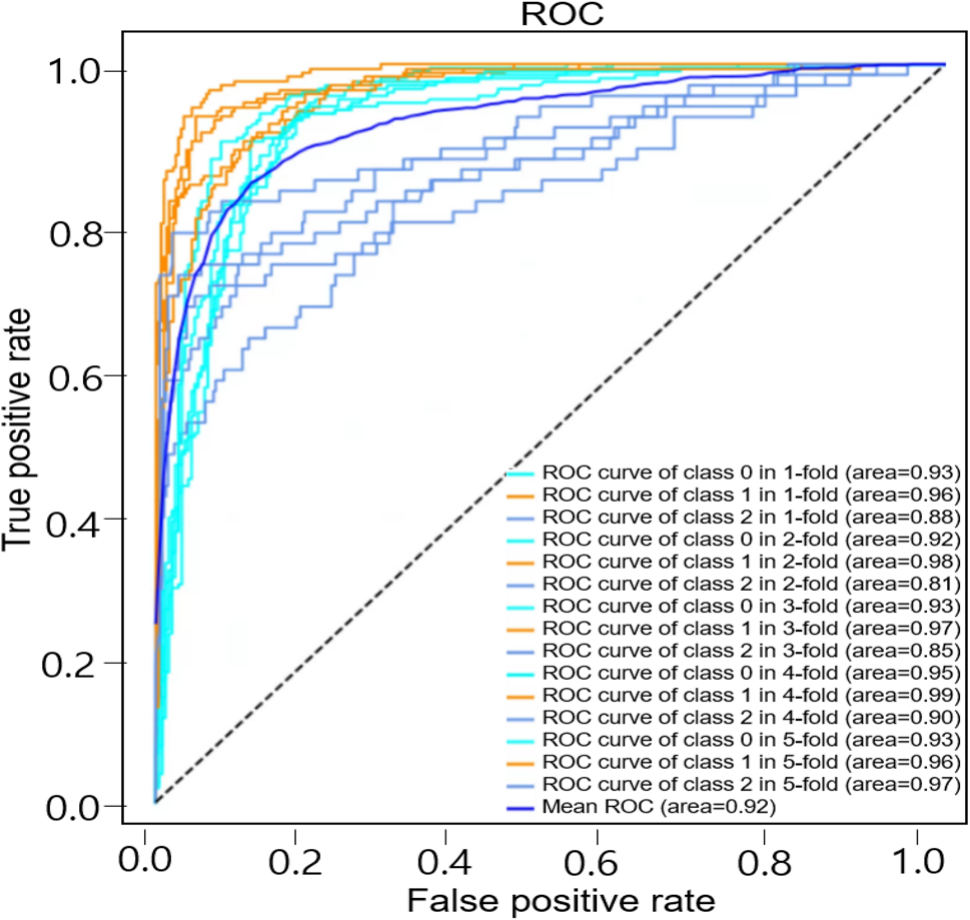
ROC of the model for classifying abnormal lung sounds into crackles, wheezes, and rhonchi. Each plot illustrates the ROC of the algorithm on the independent testing set for crackles, wheezes, and rhonchi with the mean AUC of 0.92.

Next, we categorized abnormal sounds as specific types of sounds: crackles, wheezes, or rhonchi using deep learning. The sound database included 297 crackles, 298 wheezes, and 101 rhonchi that were confirmed by specialists. The precision, recall, and F1 scores for crackles were 90%, 85%, and 87% respectively. In the case of wheezes, the precision, recall, and F1 scores were 89%, 93%, and 91%. Finally, the precision, recall, and F1 scores for rhonchi were 68%, 71%, and 69% respectively (Table 4). The average accuracy was 85.7% and the mean was AUC 0.92 (Fig. 3).

**Table 4:** The averages of Precision, Recall and F1 score in discriminating crackles, wheezes, and rhonchi.

	Precision	Recall score	F1 score
Crackles	0.90	0.85	0.87
Wheezes	0.89	0.93	0.91
Rhonchi	0.68	0.71	0.69

### Comparison of performances of different feature extractors with CNN classifier

Respiratory sounds, especially abnormal sounds, have very complicated structures with noise, and positional dependency in time. In the sound analysis, particularly mathematical point of view, its 2-D spectral-domain has more information rather than one dimensional time-series. Moreover, the deep learning structure gives an automatic feature extraction overcoming the difficulties on complicate data, especially image data. For this reason, we adopted CNN, which is a powerful method in image classification. To find out the most optimized strategy for the classification of respiratory sounds, we also compared the accuracy, precision, recall score and F1 score of each analytic method (Table 5). CNN classifier showed the best performance with VGG, especially, VGG16 rather than InceptionV3, DenseNet201, ResNet50, and ResNet101. Since VGG architecture has a better capability, especially in extracting image features for classification using transfer learning^40,41^, we adopted it for our AI models.

**Table 5:** Comparison of Performance among feature extractors with CNN classifier.

Feature extractor	Accuracy	Precision	Recall score	F1 score
InceptionV3	0.748	0.722	0.727	0.720
DenseNet201	0.786	0.749	0.735	0.729
ResNet50	0.783	0.747	0.738	0.738
ResNet101	0.809	0.787	0.766	0.769
VGG16	0.857	0.823	0.828	0.824
VGG19	0.848	0.814	0.817	0.814

Additionally, we compared the performance between CNN and SVM classifiers in order to investigate classifier dependency of feature extractor. CNN showed better performance than SVM, and VGG16 was the best classifier for both CNN and SVM. Moreover, CNN was more efficient in computation time than SVM (Table 6).

**Table 6:** Comparison of performance between CNN and SVM with feature extractors (Inception V3, DenseNet201, VGG16).

Classifier	Feature extractor	Accuracy	Precision	Recall score	F1 score
CNN	InceptionV3	0.748	0.722	0.727	0.720
	DenseNet201	0.786	0.749	0.735	0.729
	ResNet50	0.783	0.747	0.738	0.738
	ResNet101	0.809	0.787	0.766	0.769
	VGG16	0.857	0.823	0.828	0.824
	VGG19	0.848	0.814	0.817	0.814
SVM	InceptionV3	0.746	0.727	0.622	0.620
	DenseNet201	0.754	0.733	0.616	0.604
	ResNet50	0.747	0.768	0.599	0.569
	ResNet101	0.749	0.757	0.607	0.589
	VGG16	0.755	0.762	0.614	0.594
	VGG19	0.750	0.752	0.617	0.609

### Accuracy of auscultation analysis in real clinical practice

**Figure 4:**
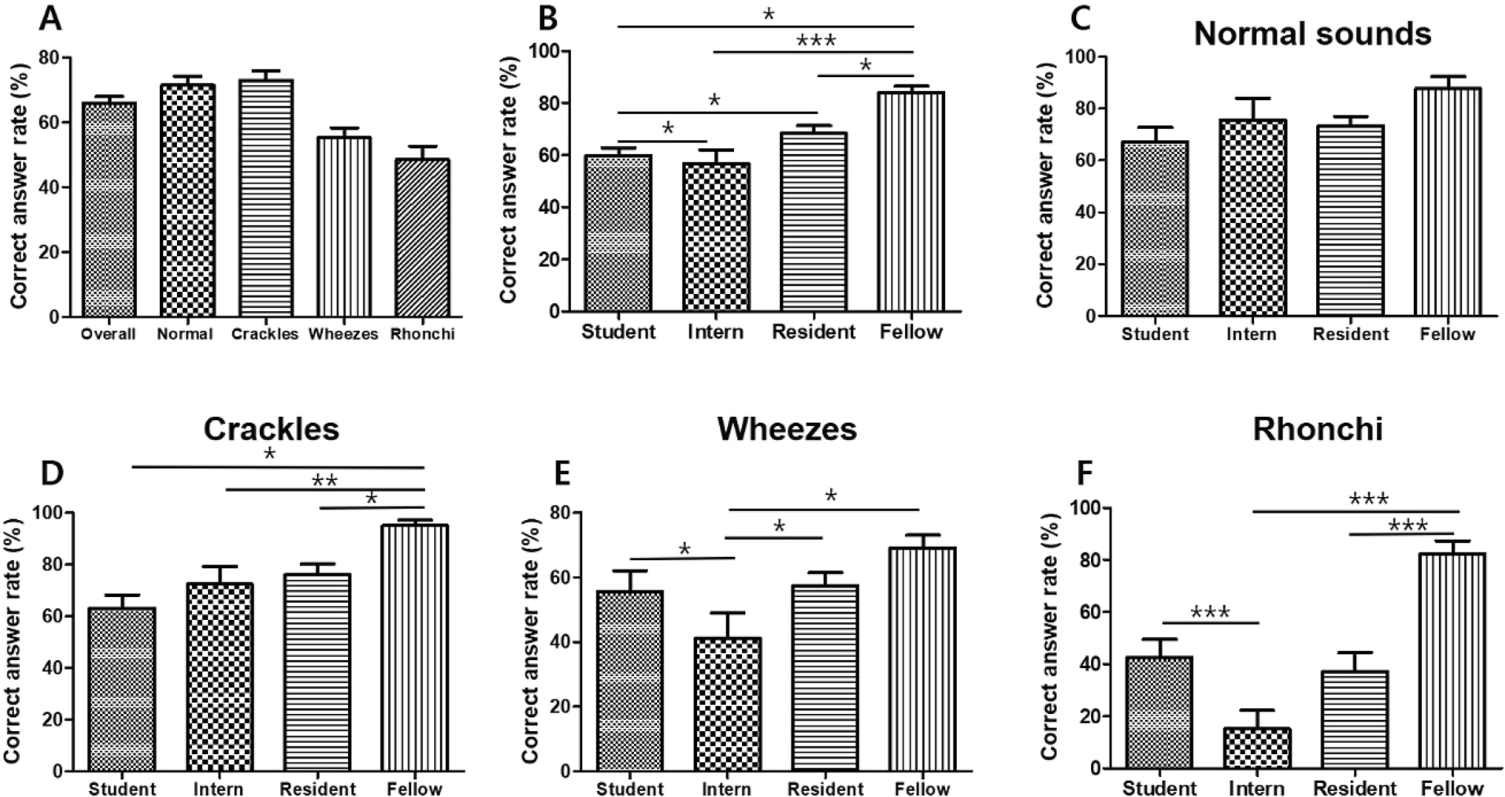
Accuracy of auscultation analysis in real clinical practice. (**A**) Mean correction answer rates for the overall sounds, normal sounds, crackles, wheezes, and rhonchi. (**B**) Mean correction answer rates of students, interns, residents, and fellows for overall sounds. (**C**) Mean correction answer rates of students, interns, residents, and fellows for normal sounds (**D**) Mean correction answer rates of students, interns, residents, and fellows for crackles (**E**) Mean correction answer rates of students, interns, residents, and fellows for wheezes. (**F**) Mean correction answer rates of students, interns, residents, and fellows for rhonchi. *p < 0.05, **p < 0.05 ***p < 0.001 (Student's t-test).

To verify the accuracy of auscultation analysis in real clinical practice and to evaluate the need for deep learning-based classification, we checked how exactly medical students, interns, residents, and fellows categorize breathing sounds (Fig. 4). We made several test sets of normal sounds and three types of abnormal lung sounds: crackle, wheezes, and rhonchi. 25 medical students, 11 interns, 23 residents, and 11 fellows of the internal medicine department of four teaching hospitals were asked to listen to the sounds and identify them. Regarding each breath sounds, the mean correct answer rates of normal sounds, crackles, wheezes, and rhonchi were 73.5%, 72.2%, 56.3%, and 41.7%, respectively. The overall correct answer rates of medical students, interns, residents, and fellows were 59.6%, 56.6%, 68.3%, and 84.0% respectively. The average correct answer rates for normal breathing were 67.1% for medical students, 75.7% for interns, 73.2% for residents, and 87.7% for fellows, while those for crackles were 62.9% for medical students, 72.3% for interns, 76.0% for residents, and 90.3% for fellows. The accuracies for wheezes were 55.6% for medical students, 41.0% for interns, 57.4% for residents, and 69.1% for fellows respectively, while those for rhonchi were 42.5% for medical students, 15.0% for interns, 37.1% for residents, and 82.2% for fellows (Fig. 4). There was no significant difference between each group in analyzing normal breathing sound, but in all three types of abnormal breathing sound, the fellows showed the highest accuracy. Among the abnormal breath sounds, interns and residents classified crackles most accurately. Rhonchi was revealed to be the most difficult sound to discriminate (Fig. 4).

## Discussion

The stethoscope has been considered an invaluable diagnostic tool for centuries^2,3^. Although many diagnostic techniques have been developed, auscultation still plays major roles^1,7^. For example, a pulmonologist can detect early-stage IPF or pneumonia based on inspiratory crackles even when the chest X-ray appears near-normal^42^. Changes in wheezes sometimes indicate the onset of asthma or COPD exacerbation. Therefore, early detection and accurate classification of abnormal breathing sounds can prevent disease progression and improve a patient’s prognosis.

Several studies have tried to automatically classify lung sounds. Chamberlain et al. classified lung sounds with a semi-supervised deep learning algorithm. The AUC were 0.86 for wheezes and 0.74 for crackles, respectively^26^. Guler et al. used a multilayer perceptron running a backpropagation training algorithm to predict the presence or absence of adventitious sounds^27^. They enrolled 56 patients and two hidden layers yielded 93.8% rated classification performance^27^.

In this study, we used deep learning for the classification of respiratory sounds. Compared with several lung sound classification studies that applied machine learning or deep learning for lung sounds classification^27,43–47^, we modified the deep learning algorithm of Bardou’s study which applied SVM^46^. In our study, we utilized the transfer learning method, which is easy, fast and able to use various features, but one has to be careful in connecting two deep learning networks, feature extractor and classifier. Moreover, there is a certain dependency between these two. We applied CNN instead of SVM because CNN is more efficient than a SVM for image classification.

Besides, our comparison of performances of different feature extractors demonstrated that CNN classifier showed much better performance with VGG, especially, VGG16 than InceptionV3 and Densenet201. The main contribution of this study is to develop the predictive model for respiratory sound classification combining pretrained image feature extractor of time-series, respiratory sound, and CNN classifier.

Our deep learning-based classification can detect abnormal lung sounds with an AUC of 0.93 and an accuracy of 86.5%. It has similar results in categorizing abnormal sounds into subcategorical sounds: crackles, wheezes, or rhonchi. Considering these are the result of analyzing the sounds recorded in a real clinical field with various noises, these are impressive results. We believe that these accuracies are adequate for primary screening and follow-up testing of patients with respiratory diseases.

Our test results showed that the auscultation accuracy of interns and residents were less than 80% in all four kinds of sounds and rhonchi was the most difficult sound to discriminate. The result of the test is not conclusive since the number of participants is small. However, it looks obvious that there are marked differences in the ability of each clinician to classify breathing sounds. This suggests that AI-assisted classification standardize the identification and categorization of breath sounds and greatly aid the diagnosis of pulmonary diseases.

There are several respiratory sounds in which two or more abnormal breath sounds are mixed. Such sounds are sometimes difficult even for experts and there may be disagreements between them. Few published studies have classified mixed abnormal breathing sounds, so research about these sounds is necessary. Also, since noises such as coughs, voices, heart sounds, and medical alarms are frequently recorded with breath sound, which reduces the accuracy of analysis, the technology for noise filtering is required.

## Conclusion

**Figure 5:**
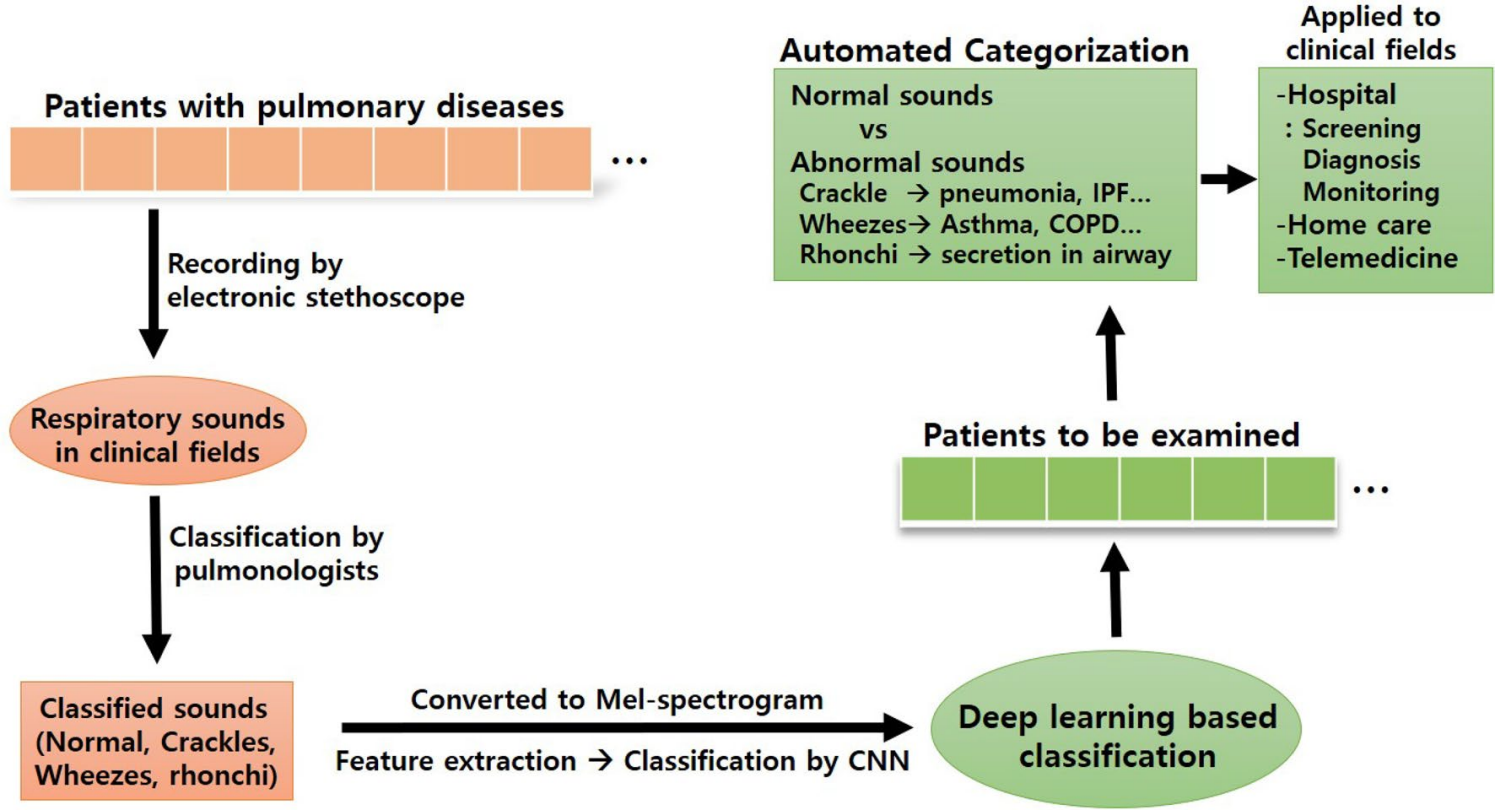
Summary of deep learning assisted classification of respiratory sounds. Respiratory sounds were corrected from the patients with pulmonary diseases. The sounds were validated and classified by pulmonologists. The sounds were converted to Mel-spectrogram and features were extracted by VGG16 (transfer learning). Respiratory sounds were classified by CNN. Deep learning-based classification of respiratory sounds can be helpful for screening, monitoring, and diagnosis of pulmonary diseases.

We found that our deep learning-based classification could classify the respiratory sounds accurately. Utilizing the transfer learning method, combining pre-trained image feature extraction from respiratory sound and CNN classification, worked well and was helpful for improving the classification accuracy. Though the analysis of mixed abnormal sounds and filtering noises remain challenging, recent innovations in analytic algorithm and recording technology will accelerate the advance of respiratory sound analysis more rapidly. Soon, deep learning-based automated stethoscope is expected to be used in telemedicine and home care (Fig. 5).

## Methods

### Patient selection and data collection

Patients who visited an outpatient clinic or were hospitalized at Chungnam National University Hospital, regardless of the presence or type of respiratory diseases were enrolled from April 2019 to December 2020. The recording was proceeded in the actual clinical field (outpatient clinic, hospitalization room, emergency room, intensive care unit). Lung sounds were obtained from two to six sites of the posterior thorax using a Littman 3200 electronic stethoscope, downloaded to a computer, and converted into “wave” files. All sounds were carefully checked and validated by three pulmonologists. All patients gave written informed consent, and we obtained human research ethics committee approval of Chungnam National University Hospital Institutional Review Board (No. 2020-10-092). All methods were performed in accordance with the relevant guidelines and regulations. We recorded 2840 sounds and made a respiratory sound database containing 1222 normal breath sounds, 297 crackles, 298 wheezes, and 101 rhonchi.

### AI models with transfer learning and CNN

#### Overview of AI models

**Figure 6:**
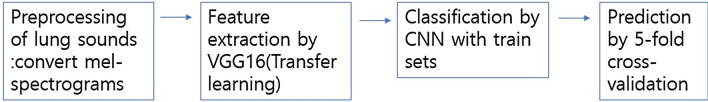
Overview of our AI models.

Lung sounds were converted to Mel-spectrograms and features were extracted by VGG16. CNN was applied for the classification and fivefold cross-validation was used for prediction (Fig. 6).

#### Preprocessing of lung sounds

**Figure 7:**
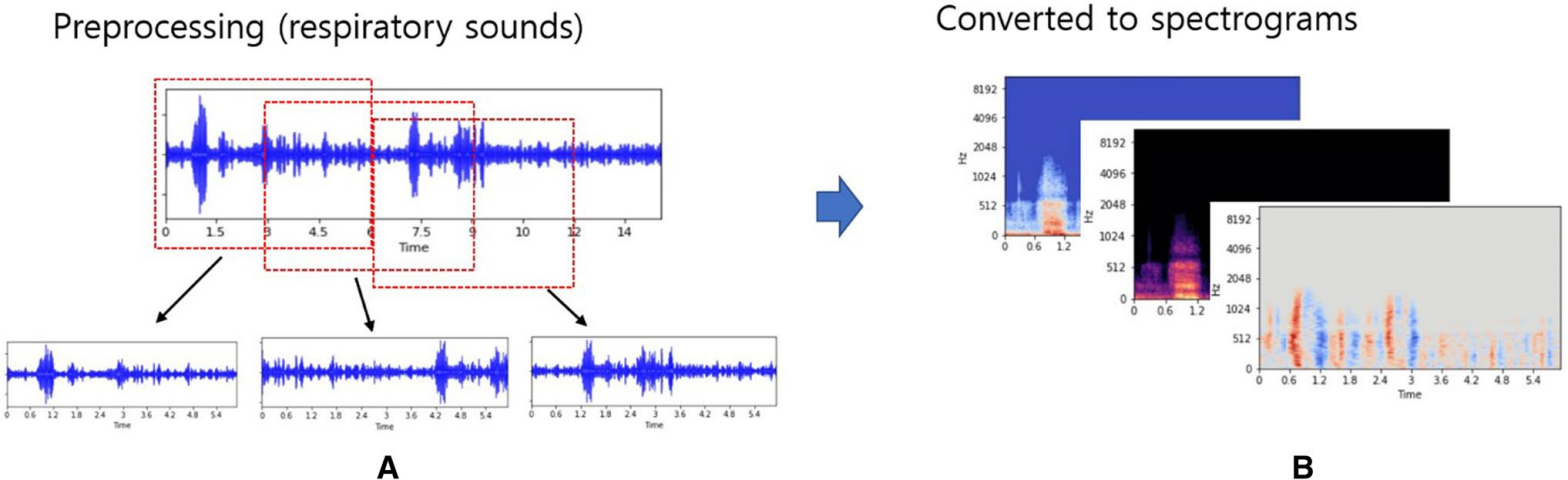
Process to obtain spectrograms. (**A**) Given lung sound, dividing lung sound files with overlapping during 3 s (**B**) Obtaining three types of Mel-spectrograms with log-scale.

Recorded sounds were ranged from a few seconds to several tens of seconds. We divided them into 6 s each with 50% overlapping. For example, the audio file is a 14.5-s audio file of wheezing, which is divided into 3 cycles according to the start and end times (Fig. 7). And, to process the feature extraction and use the 3-dimensional input data, we used Mel-spectrogram, average of harmonic and percussive Mel-spectrogram, and the derivative of Mel-spectrogram using the Python library librosa^47^.

#### Feature extractor and classification

We thought at least two or three cycles of respiratory sounds are needed for accurate analysis of lung sounds. Approximately, normal respiratory rate is 15–20 per one minute (three–four seconds per one respiratory cycle) and it tends to be more rapid at pathologic conditions. So, after testing several options, we finally have decided six seconds as the length of the respiratory sound.

**Figure 8:**
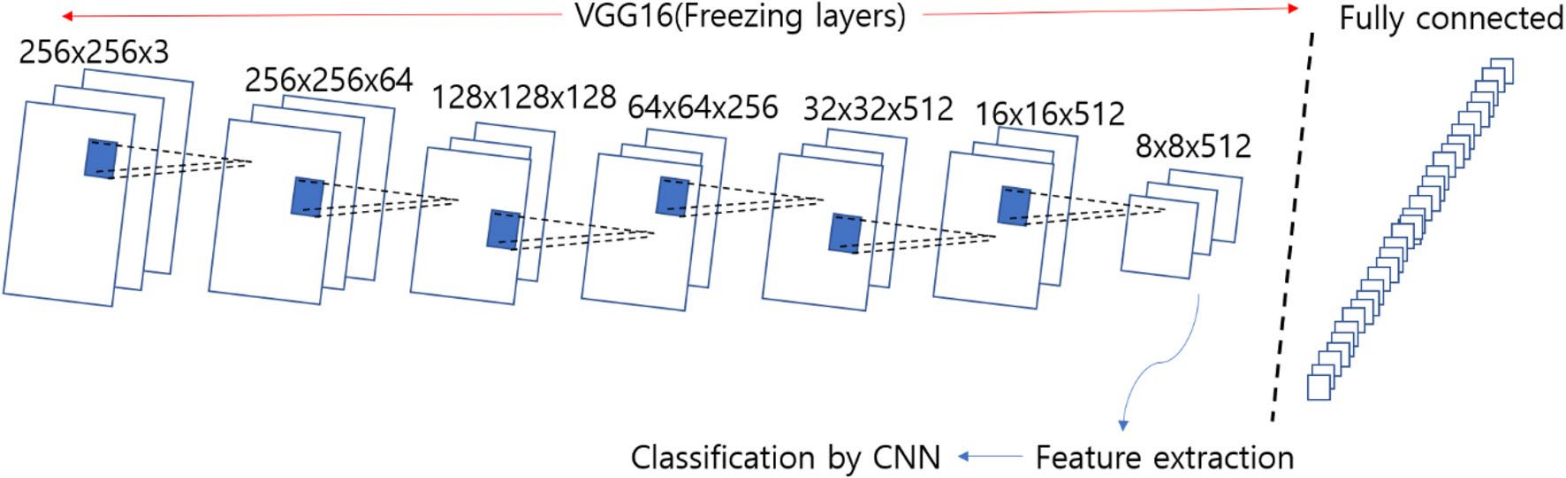
VGG16 architecture for our model. The input size for our model is 256 × 256. We freeze all layers in VGG16 without fully-connected layer, and with extracting features, we classified the respiratory sounds.

We used pre-trained models VGG16 as feature extractors in transfer learning, which was built by Karen Simonyan^48,49^. VGG16 is a model with 16 layers trained on fixed-size images and the input is processed through a set of convolution layers that use small-size kernels with a receptive field 3 × 3. The default input size of VGG16 is 224 × 224, but the input size for our model is 256 × 256 (Fig. 8). We used weights pre-trained on ImageNet by freezing all the five convolution blocks without fully-connected layer, and predicted the test sets with simple CNN with only one-layer.

#### Evaluation of our models

**Figure 9:**
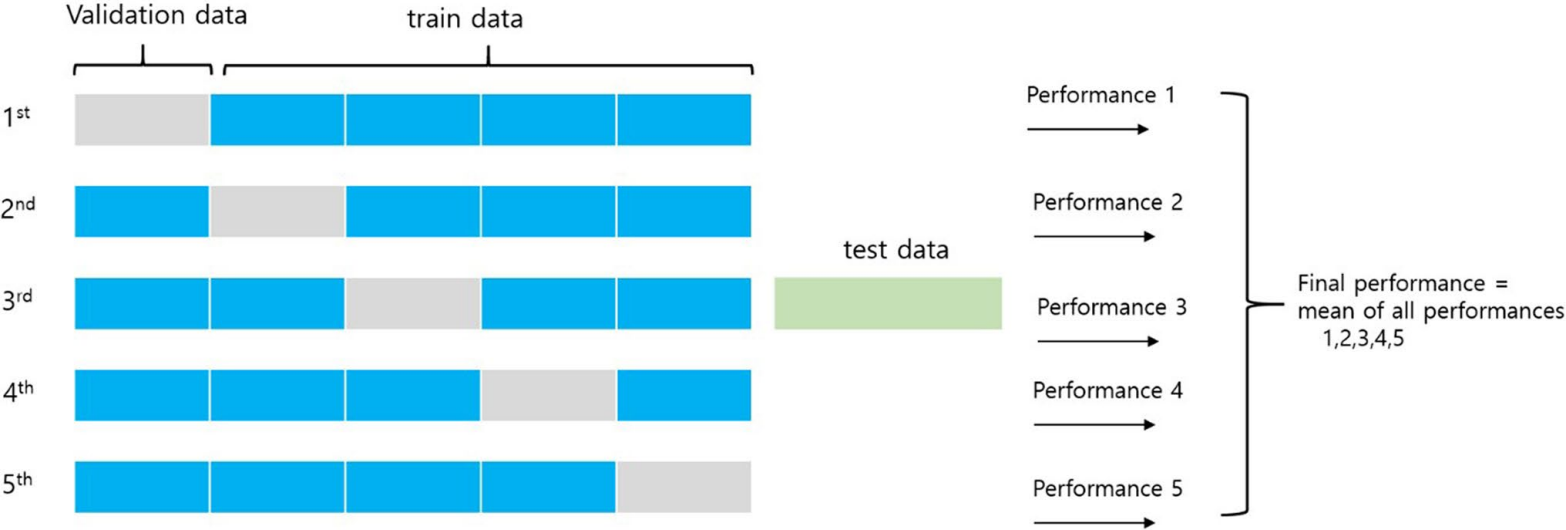
5-folds cross validation. Trying 5 iterations and getting final results with mean of all performances.

To avoid overfitting, we utilized the fivefold cross-validation method^34^ (Fig. 9). The dataset has been chosen randomly to split into 80% training set and 20% test set, and 20% of training set is used for validation. The main idea of the fivefold cross validation is to split the training set into 5 partitions. Each time one of the 5 partitions are used for validating the model and the other 4 partitions are used for training the model. So, each instance in the data set is used once in testing and 4 times in training. All results of the different metrics are then averaged to return the result. From results by our models, we obtained accuracy, precision, recall score and ROC curve.

#### Statistical analysis

All values are presented as means ± standard deviation (SD). Significant differences were determined using GraphPad 5 software. The Student’s t-test was used to determine statistical differences between two groups. The receiver operating characteristic curve was plotted and the area under the curve was calculated with the 95% confidence intervals.
